# The Role of c-Met as a Biomarker and Player in Innate and Acquired Resistance in Non-Small-Cell Lung Cancer: Two New Mutations Warrant Further Studies

**DOI:** 10.3390/molecules24244443

**Published:** 2019-12-04

**Authors:** Nele Van Der Steen, Karen Zwaenepoel, Giulia Mazzaschi, Rosa A. Luirink, Daan P. Geerke, Ken Op de Beeck, Christophe Hermans, Marcello Tiseo, Paul Van Schil, Filip Lardon, Paul Germonpré, Christian Rolfo, Elisa Giovannetti, Godefridus J. Peters, Patrick Pauwels

**Affiliations:** 1 Center for Oncological Research, University of Antwerp, Universiteitsplein 1, 2610 Antwerp, Belgium; nelevandersteen@gmail.com (N.V.D.S.); Karen.Zwaenepoel@uza.be (K.Z.); ken.opdebeeck@uantwerpen.be (K.O.d.B.); Christophe.Hermans@uantwerpen.be (C.H.); filip.lardon@uantwerpen.be (F.L.); Paul.Germonpre@azmmsj.be (P.G.); Christian.Rolfo@umm.edu (C.R.); 2 Department of Pathology, Antwerp University Hospital, Wilrijkstraat 10, 2650 Antwerp, Belgium; 3 Department of Medical Oncology, VU University Medical Center, CCA 1.42, De Boelelaan 1117, 1081 HV Amsterdam, The Netherlands; gj.peters@amsterdamumc.nl; 4 Department of Medicine and Surgery, University of Parma and Medical Oncology Unit, University Hospital of Parma, 43126 Parma, Italy; giulia.mazzaschi@studenti.unipr.it (G.M.); mtiseo@ao.pr.it (M.T.); 5 AIMMS Division of Molecular Toxicology, Department of Chemistry and Pharmaceutical Sciences, Faculty of Sciences, VU University Amsterdam, De Boelelaan 1108, 1081 HZ Amsterdam, The Netherlands; r.a.luirink@vu.nl (R.A.L.); D.p.geerke@vu.nl (D.P.G.); 6 Center of Medical Genetics, University of Antwerp and Antwerp University Hospital, Prins Boudewijnlaan 43, 2650 Antwerp, Belgium; 7 Department of Thoracic and Vascular Surgery, University Hospital, Wilrijkstraat 10, 2650 Antwerp, Belgium; paul.vanschil@uza.be; 8 Department of Pneumology, AZ Maria Middelares, Kliniekstraat 27, 9050 Gentbrugge, Belgium; 9 Phase I-Early Clinical Trials Unit, Oncology Department, Antwerp University Hospital, Wilrijkstraat 10, 2650 Antwerp, Belgium; 10 Thoracic Medical Oncology, Marlene and Stewart Greenebaum Comprehensive Cancer Center, University of Maryland School of Medicine, Baltimore, MD 20742, USA; 11 Cancer Pharmacology Lab, AIRC Start-Up Unit, Fondazione Pisana per la Scienza, Via Giovannini 13, San Giuliano Terme, I-56017 Pisa, Italy; 12 Department of Biochemistry, Medical University of Gdansk, 80-844 Gdansk, Poland

**Keywords:** NSCLC, c-Met, EGFR, biomarkers

## Abstract

The c-Met receptor is a therapeutically actionable target in non-small-cell lung cancer (NSCLC), with one approved drug and several agents in development. Most suitable biomarkers for patient selection include c-Met amplification and exon-14 skipping. Our retrospective study focused on the frequency of different c-Met aberrations (overexpression, amplification and mutations) in 153 primary, therapy-naïve resection samples and their paired metastases, from Biobank@UZA. Furthermore, we determined the correlation of c-Met expression with clinicopathological factors, Epidermal Growth Factor Receptor (EGFR)-status and TP53 mutations. Our results showed that c-Met expression levels in primary tumors were comparable to their respective metastases. Five different mutations were detected by deep sequencing: three (E168D, S203T, N375S) previously described and two never reported (I333T, G783E). I333T, a new mutation in the Sema(phorin) domain of c-Met, might influence the binding of antibodies targeting the HGF-binding domain, potentially causing innate resistance. E168D and S203T mutations showed a trend towards a correlation with high c-Met expression (*p* = 0.058). We found a significant correlation between c-MET expression, EGFR expression (*p* = 0.010) and *EGFR* mutations (*p* = 0.013), as well as a trend (*p* = 0.057) with regards to TP53 mutant activity. In conclusion this study demonstrated a strong correlation between EGFR mutations, TP53 and c-Met expression in therapy-naïve primary resection samples. Moreover, we found two new c-Met mutations that warrant further studies.

## 1. Introduction

The therapeutic landscape of non-small-cell lung cancer (NSCLC) harboring oncogenic driver alterations has revolutionized by the introduction of tyrosine kinase targeted inhibitors (TKIs), such as Epidermal Growth Factor Receptor (EGFR) [1], and Anaplastic Lymphoma Kinase (ALK) TKIs [2]. Currently, there are several biomarker-defined patient subgroups, in which treatment with specific TKIs have superior clinical outcomes compared to standard conventional cytotoxic chemotherapy. However, still only a limited fraction of NSCLC patients may benefit from these agents. Thus, the challenge now faced is to identify the patient- and tumor-specific biomarkers holding the most promise to screen and select appropriate patients for TKI treatment.

The L858R mutation and exon 19 deletion in EGFR as well as Anaplastic Lymphoma Kinase (ALK) translocation have been demonstrated as reliable biomarkers for the response to EGFR [1] and ALK inhibitors [2], respectively. Nonetheless, in most cases, acquired resistance against TKIs occurs after an average of one year, leading to renewed tumor growth and progression, suggesting specific pathogenetic mechanisms, e.g., c-Met amplification [3,4,5].

For c-Met, it has been established that its aberrations (in particular, gene amplification or c-Met exon 14 skipping mutations) represent oncogenic drivers [6] and resistance mechanisms against EGFR-TKIs [5]. In cancer, the activation of c-Met has been associated with invasive growth [7], tubulogenesis [8], angiogenesis [9] and the induction of Epithelial Mesenchymal Transition (EMT) [10]. The signaling pathways of c-Met and EGFR are strictly intertwined, since EGFR activation leads to increased activation of c-Met, and vice versa [11,12,13]. Dysregulation of the c-Met pathway in lung cancer occurs through a variety of mechanisms including gene mutation, amplification, rearrangement, and protein overexpression. The overexpression of c-Met is found in nearly 50% of patients [14,15,16], whereas c-Met amplification is detected in only 2%–3% of EGFR-TKI naïve patients [15,16]. In contrast, approximately 10% of EGFR-TKI treated patients show acquired c-Met amplification as a resistance mechanism [4]. Patients with a high level of c-Met amplification (c-Met/CEN7 of ≥5) or c-Met exon 14 skipping respond well to c-Met TKIs [17,18], likely because exon 14 contains Tyr1003, which is necessary for the ubiquitylation and breakdown of c-Met [19]. Finally, mutations of c-Met other than exon 14 skipping have been reported [20,21] to be able to affect c-Met activity, although the response to c-Met-TKIs is still unknown.

Several clinical trials with c-Met TKIs (crizotinib [18,22], cabozantinib [23], capmatinib [24], tepotinib [25], tivantinib [26]) or monoclonal antibodies (onartuzumab [27,28], emibetuzumab, rilotumumab [29], ficlatuzumab [30]) have been completed or are in progress. Moreover, given the significant crosstalk between the c-Met and EGFR pathways [11,12], the therapeutic strategy to combine c-Met and EGFR inhibitors has been repeatedly explored, and most trials are still ongoing [31]. The choice of this approach is partially based on the documented synergy of c-Met and EGFR in driving oncogenesis in both EGFR wild type (WT) and mutant lung cancer models, in the setting of acquired resistance to EGFR-TKIs.

In this context, the c-Met status is usually determined by immunohistochemistry (IHC) and not by fluorescence in situ hybridization (FISH) or mutation analysis. Therefore, we aimed to determine the correlation between c-Met expression and other c-Met aberrations (amplification, exon 14 skipping, mutations) or EGFR mutation status.

With regards to EGFR-targeted agents, exon 19 deletions and exon 21 L858R substitutions, termed classic mutations, represent the most common genetic alterations, accounting for approximately 90% mutations in NSCLC adenocarcinomas and resulting in a high sensitivity to the first generation of EGFR-TKIs [1]. Conversely, other uncommon EGFR mutations, including G719X, S768I, L861Q, exon 20 insertions and complex mutations, are able to determine more efficient responses to second- and third-generation TKIs compared to first-generation ones and have been the target of various drugs (i.e., poziotinib) [32,33].

The main resistance mechanism to the first- and second-generation TKIs is the occurrence of the T790M mutation detected in more than 50% of the patients after disease progression [34]. Osimertinib (AZD9291), a third-generation EGFR inhibitor, selectively blocks the activated EGFR mutant cells carrying the T790M-resistant mutation [1]. Nonetheless, several osimertinib-resistant processes have been demonstrated, among which the acquired EGFR C797S mutation represents the most frequent [35,36,37]. Currently, the fourth generation of EGFR inhibitors is under intense development to overcome this resistance mechanism [38]. Moreover, the activation of c-Met is considered as an important component that negatively affects EGFR-inhibitor effectiveness, due to the previously mentioned intertwining of both pathways [11].

TP53 is a tumor suppressor that is mutated in 50% of lung cancers [39]. Three types of mutations can be distinguished: mutations that change the DNA interaction site of p53 (e.g., R273, R248), mutations that change the protein structure (G245, R249) and silent mutations that might cause aberrant splicing (E204) [40]. It has been shown that p53 represses c-Met transcription by binding the c-Met promotor [41]. This leads to c-Met overexpression in cases of mutant/deficient TP53 [41,42]. Mutant TP53 (273H or 175H) promotes c-Met receptor recycling, thus increasing the sensitivity of the cells to HGF and leading to increased invasion and cell scattering [43]. In the case of EGFR-mutated NSCLC, the co-occurrence of TP53 mutations correlates with a shorter response time to EGFR targeted therapies [44]. In this study, 88% of short-term responders presented with a co-occurring TP53 mutation, in comparison to 18% in the long-term responders group.

In the present study, we aimed to investigate the c-Met-biomarker status of targeted therapy-naïve NSCLC patients, thus assessing the patient population potentially eligible for c-Met-TKIs as a first-line treatment. Subsequently, given the intertwining of the c-Met, EGFR and TP53 pathways, we addressed our efforts to determine both the gene- (c-Met, EGFR, TP53) and protein-expression status (c-Met and EGFR) of these targets in a TKIs-naïve NSCLC patient population, and correlate these different results with each other.

Of note, these studies detected five different mutations of c-Met: three (E168D, S203T, N375S) previously described, and two never reported (I333T, G783E). In particular, I333T, a new mutation in the Sema (phorin) domain of c-Met, might influence the binding of antibodies targeting the HGF-binding domain, such as onartuzumab, potentially causing innate resistance. These results support that a deep tumor profiling will be an important part of clinical management of NSCLC patients, when using new c-Met inhibitors, and warrant further investigation.

## 2. Results

### 2.1. c-Met Expression and Gene Amplification

The expression level and amplification status of c-Met were determined in 153 primary formalin fixed paraffin embedded (FFPE) NSCLC tumors from EGFR-TKI naïve patients. In total, 151/153 of the IHCs were eligible for scoring. Forty-eight percent (72/151) of samples showed high c-Met expression (2+/3+).

This expression was correlated with the tumor histology (*p* = 0.016), with a high c-Met expression in 56% of adenocarcinomas versus 35% of squamous and 9% of large cell carcinomas or not otherwise specified (NOS). The expression was independent of smoking history (*p* = 0.725), gender (*p* = 0.497), tumor differentiation (*p* = 0.160), invasiveness (*p* = 0.377), tumor status/T (*p* = 0.544), lymph node status/N (*p* = 0.061) and metastatic status/M (*p* = 0.380). The Kaplan-Meier curve shows no influence on the survival time (*p* = 0.785) (Supplementary Figure S1).

A total of 108 out of 153 samples for chromogenic in situ hybridization (CISH) were interpretable, out of which only four (3.7%) displayed c-Met amplification: ratios c-Met/CEN7 4.54, 2.61, 2.05 and 2.00. Only the sample with a ratio of 4.54 showed focal amplification of c-Met. Half of the c-Met amplified samples, including the sample with a ratio of 4.54, showed a high c-Met expression (3+), the others had a score of 0. The internal controls were positive in all samples.

### 2.2. c-Met Primary Tumor Versus Metastasis

Forty-one paired metastases (27 synchronous and 14 metachronous) were tested. The Cohen’s kappa test (high: (3+ and 2+) vs low (1+ and 0)), with a kappa-value of 0.430, showed a moderate agreement (95% CI: 0.146–0.714; *p* = 0.006) in c-Met expression in primary tumor samples vs metastases.

There was no significant correlation (*p* = 0.147) between the c-Met expression and the timing of the metastasis (synchronous/metachronous). One patient showed c-Met amplification in the primary tumor (ratio 2.05), but not in the synchronous lymph node metastasis. From the other c-Met-amplified tumors, no metastatic tissue was available. Another patient showed amplification (ratio 2.31) in a metachronous liver metastasis but not in the primary tumor itself.

### 2.3. Correlation between c-Met and EGFR

EGFR-IHC and mutational analysis were performed in 61/104 adenocarcinomas, with available specimens. In total, 31/61 (51%) were positive (2+/3+) for EGFR-IHC, while 14/45 (31%) had EGFR mutations: L858R (eight cases), exon 19 deletion (three cases) and exon 20 insertion (three cases). This high percentage might be explained by the high percentage of non-smokers in this cohort of patients.

A significant correlation (*p* = 0.010) between EGFR and c-Met expression was found. Here, 20% of samples with EGFR-IHC 0 show high c-Met expression, versus 35% of EGFR 1+, 84% of EGFR 2+ and 92% of EGFR 3+ samples.

**Figure 1:**
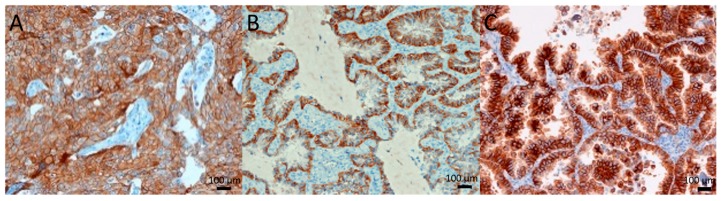
c-Met-IHC of EGFR-mutant NSCLC. (**A**) L858R mutation, (**B**) exon 19 deletion, (**C**) exon 20 insertion. All tumors with EGFR mutants showed moderate to high c-Met expression (2+–3+).

In EGFR-mutated samples, a high c-Met expression (2+ and 3+) was found in all 14/14 samples (Figure 1), versus 16/31 samples (52%) in the EGFR-WT group. No significant correlation was found (*p* = 0.436) between the types of EGFR mutation and c-Met expression. The two EGFR-tested c-Met-amplified samples were EGFR-WT. The sample with a ratio of 2 showed an EGFR expression of 1+, whereas the sample with a ratio of 4.54 showed an expression of 2+.

### 2.4. c-Met and TP53 Mutations

Exon 14 skipping of c-Met was found in two patients, with an allelic ratio of 12% and 39%. Both samples showed a high c-Met expression. The first sample also had a co-occurring c-Met S203T mutation.

In 14/69 sequenced primary tumor samples, five non-synonymous mutations in c-Met (Table 1) were found. Four mutations were in the Sema domain and one (G783E) in the Ig3 domain. The protein database (PDB) ID 1SHY [45] shows the Sema domain in complex with HGF. PDB 4K3J [46] shows the Sema domain in complex with HGF-β and onartuzumab. N375S, E168D and S203T have been described previously [47,48].

**Figure 2:**
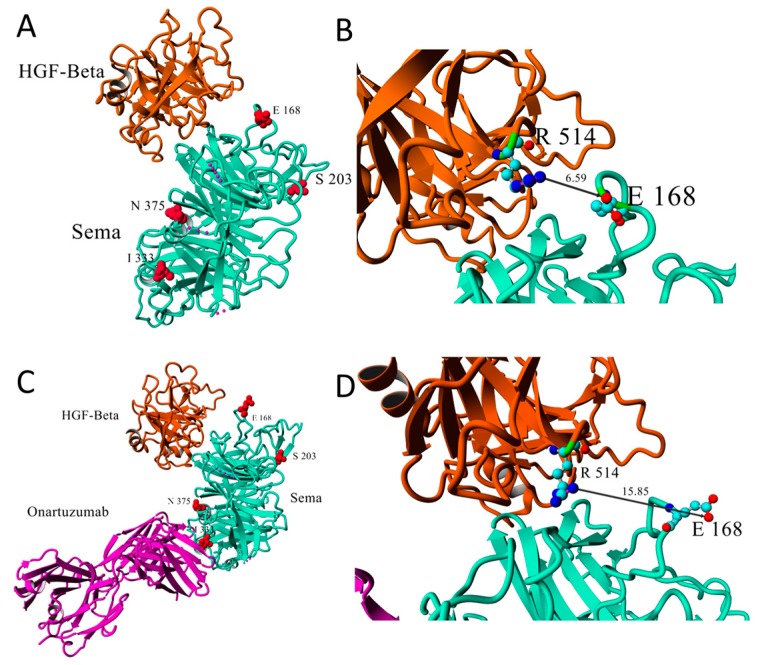
(**A**) Crystal structure reported by Stamos et al. [45] (PDB ID 1SHY) of c-Met domain Sema in complex with the β domain of Hepatocyte growth factor HGF. HGF-β is depicted in brown, the c-Met Sema domain is depicted in cyan. Sema residues are highlighted in red and in ball-representation. (**B**) Zoom-in of the HGF-Sema binding region in (A), with Sema residue E168 and HGF-β residue R514 highlighted in ball-and-stick and CPK-color representation. The distance between the closest E168 carbonyl oxygen and R514 guanidium nitrogen is given in Å. (**C**) Crystal structure reported by Merchant et al. [46] (PDB ID 4K3J) of c-Met domain Sema in complex with the β domain of Hepatocyte growth factor HGF and with onartuzumab. Onartuzumab is depicted in magenta. (**D**) Zoom-in of the HGF-Sema-binding region in (**C**), see (B). All structures are displayed with Yasara View (yasara.org)

N375S is a germline mutation [48] within the HGF-α binding site (Figure 2A,C). Krishnaswamy et al. [47] reported a lower affinity of c-Met for HGF as measured in ELISA-based assays. This lower affinity was due to the loss of a hydrogen bond between residue 375 and the adjacent Arg280. The N375S mutation is associated with a decreased sensitivity of the ATP-competitive inhibitor SU11274.

The germline E168D mutation leads to a higher affinity for HGF and higher susceptibility of c-Met for SU11274 [47]. The E168D mutation is at the interface of the Sema-HGF-β complex (Figure 2A), and the amino acid is in close contact with residue R514 of HGF-β (Figure 2B). However, the distance between the closest E168 carbonyl oxygen and R514 guanidium nitrogen varies between the 1SHY and 4K3J crystal structures (from 6.59 Å to 15.85 Å, respectively, Figure 2B,D).

Although the S203T mutation is registered in ClinVar, no further studies assessed its role in c-Met signaling. This mutation was reported as benign and germline. We have mapped the I333T, G783E and S203T mutations onto c-Met. In the 4K3J complex, the locations of onartuzumab and N375S are both close to I333 (Figure 2C), suggesting that the I333T mutation may affect the binding of the Sema-domain to onartuzumab. This conclusion does not change when considering the structure of mutant I333T as predicted by the I-TASSER webserver (https://zhanglab.ccmb.med.umich.edu/I-TASSER/), in which the position of this residue and the structure of the interface region are predicted to be not substantially unaffected by the isoleucine to threonine mutation (see Figure S2 of the Supplementary Material).

Mutations S203T (Figure 2A,C) and G783E are far from the Sema-HGF binding interfaces, and will probably not affect HGF binding to Sema or c-Met susceptibility for SU11274.

There was no association between the c-Met expression and the mutation status (*p* = 0.9). When comparing the different mutations separately, there was a strong trend (*p* = 0.058) towards an association between two c-Met mutations and c-Met expression. Here, two patients with an E168D mutation both showed 3+ c-Met expression. The S203T mutation was found in seven patients, one showed no c-Met expression, one showed 1+ and five showed 2+ expression. All other patients with c-Met mutations showed no to low expression. c-Met mutations did not correlate with EGFR status.

Inactive TP53 mutations were found in 43% of patients (30/69) (Table 2). The TP53 mutations and the c-Met expression were not correlated (*p* = 0.381). The TP53 mutations were divided into partial-active and non-functional groups according to the TP53 Mut Assessor tool of the TP53 database [49], based on the percentage of remaining activity. A remaining activity of 10% was used as cut-off.

When comparing c-Met expression with TP53, a strong trend (*p* = 0.057) was found, with 72% of p53 non-functional samples presenting with a high c-Met expression as compared to 36% of p53 partially-functional samples. Invasiveness [49] and TP53 functionality were not correlated (*p* = 0.131). Here, 75% of tumors with non-functional TP53 showed an invasive phenotype, versus 40% of tumors with partially active TP53.

## 3. Discussion

Heterogeneity in c-Met staining [50] was documented in the majority of the samples in the present study. Fifty percent of tumors showed a high expression of c-Met, which is in accordance with previous studies [16,50]. Along the same line, the percentage of c-Met amplification was in agreement with the literature [51], ranging from 2% to 10% [16]. There was a significant correlation of c-Met expression and tumor histology, with adenocarcinoma showing a high c-Met expression (56%) more frequently as compared to squamous carcinoma (35%) and large cell carcinoma or NOS (9%), which is in consistent with previous reports [16]. Although we divided histology into three subtypes (adenocarcinoma, squamous carcinoma and large cell lung cancer or NOS) in contrast to the usual squamous and non-squamous division [52], this correlation remains significant.

We also found a trend towards a correlation with positive lymph node involvement. This evidence might suggest that tumor cells activate c-Met during the formation of lymph node metastasis, but subsequently rely on other signaling pathways to develop distant metastases. Additionally, the role of HGF and c-Met in lymphangiogenesis [8,9] might contribute to its influence during the formation of lymph node metastases. In contrast to other studies [16], we did not find a prognostic role for c-Met expression. However, the retrospective character of this study and the wide range of different treatment schedules of patients undoubtedly played a role.

The amplification of c-Met was heterogeneous; therefore, we opted to score 10^5^ cells. Previous studies showed a distinction between a low, moderate and high amplification in response to c-Met-TKIs [18]. Here, only in the sample with a ratio of 4.54 foci of the amplified c-Met gene were visible, whereas for the lower amplified samples, with a ratio of around 2, this was not the case. Due to the retrospective character of this study, none of the patients received c-Met-TKI treatment. However, we hypothesize that clusters of the c-Met gene might serve as an extra criterion for patient selection, although further studies are required to confirm this hypothesis.

Based on the premise that c-Met signaling is strongly intertwined with EGFR signaling [11,12], the documented strong correlation of c-Met with both EGFR expression and EGFR mutations is not surprising. Nonetheless, this finding is remarkable, since these tumors have never been treated with EGFR-TKIs. Both sensitizing EGFR mutations (L858R or exon 19 deletion) and resistance mutations (exon 20 insertion) were associated with a high c-Met expression, with all EGFR mutant tumors showing a high c-Met expression. This suggests that c-Met signaling might already play a role in tumor growth before EGFR-TKI treatment, not only acting as a resistance mechanism against EGFR-TKIs. It may be conceivable that the high expression of c-Met in EGFR-mutant patients can also be implicated in unpredicted low responses or early acquired resistance of NSCLC patients to EGFR-TKIs. In this case, the activation of the c-Met receptor represents a crucial factor, which warrants further study to determine the phosphorylation status of c-Met. Thus, in cases where a higher c-Met expression is coupled to a higher activation of c-Met, this opens the door to combine EGFR and c-Met inhibitors, including the screening for c-Met activation together with EGFR mutations, as previously postulated [53,54].

TP53 regulates c-Met expression by binding the c-Met promotor. Downregulation or inactivation of p53 protein leads to an increased c-Met expression and rescue of p53 leads to a decrease in c-Met expression [41,42]. Mutations in TP53 can be divided into two groups: partially functional p53 or non-functional p53. There was a lower increase in c-Met expression in cases of partial activity of p53 as compared to non-functional p53. This p53-c-Met relation has been demonstrated in the cell lines of different tumor types [41,42,55]. In our study, 50% of NSCLC carried TP53 mutations [39], while approximately the same percentage presented with c-Met overexpression. This link could suggest that c-Met expression is mostly a consequence of transcriptional regulation and not a sign of c-Met addiction as with c-Met amplification or c-Met exon 14 skipping. Since the activation of c-Met promotes metastasis, it was not unexpected that a part of primary tumors showed an increase in c-Met expression in the paired metastases. However, some metastases from primary tumors with high c-Met expression showed only a low c-Met expression in the metastasis. Several reasons might cause this phenomenon. Firstly, there is no method to determine the exact timing of the metastasis of the tumor. It is possible that c-Met is upregulated during the process of metastasis and downregulated immediately after, resulting in a lower expression of c-Met. Also, the micro-environment might play a role with the amount of HGF, thus influencing the activity and presence of c-Met [56].

Five non-synonymous mutations in c-Met have been identified: N375S, E168D, S203T, I333T and G783E, of which S203T and I333T are two newly reported mutations in the ligand-binding domain. Modelling in silico revealed that S203T is located outside the HGF-binding domain and probably may not lead to an altered functioning of the receptor. I333T is located in the HGF-binding domain and might influence HGF affinity, but functional studies are needed to confirm this. In addition, I333T is located in the interface of the Sema-domain and onartuzumab [46]. Although further testing is needed to confirm this, the I333T mutation may affect the binding of anti-c-Met antibodies targeting the HGF-binding domain and confer innate resistance to them by preventing them from binding to the receptor. These findings on novel c-Met mutations suggest that a robust tumor profiling will be an important part of patient care, when using new c-Met inhibitors.

## 4. Materials and Methods

### 4.1. Patients

A total of 153 NSCLC FFPE samples of Caucasian treatment-naïve patients who underwent resection between 2004 and 2013 at the University Hospital Antwerp and the Onze-Lieve-Vrouw-Hospital Aalst were examined in a retrospective study (Table 3). The patients were treated in local hospitals according to the preference of the local physician. All histological subtypes and stages of NSCLC were included. The population was enriched with patients with EGFR-activating mutations. If paired metastases were available, these were included in the study.

Ethical approval was obtained: B300201316249 in Antwerp University Hospital and B300201317801 in the Onze-Lieve-Vrouw-Hospital Aalst. The human biological material used in this publication was provided by Biobank@UZA [57].

### 4.2. IHC and CISH

**Figure 3:**
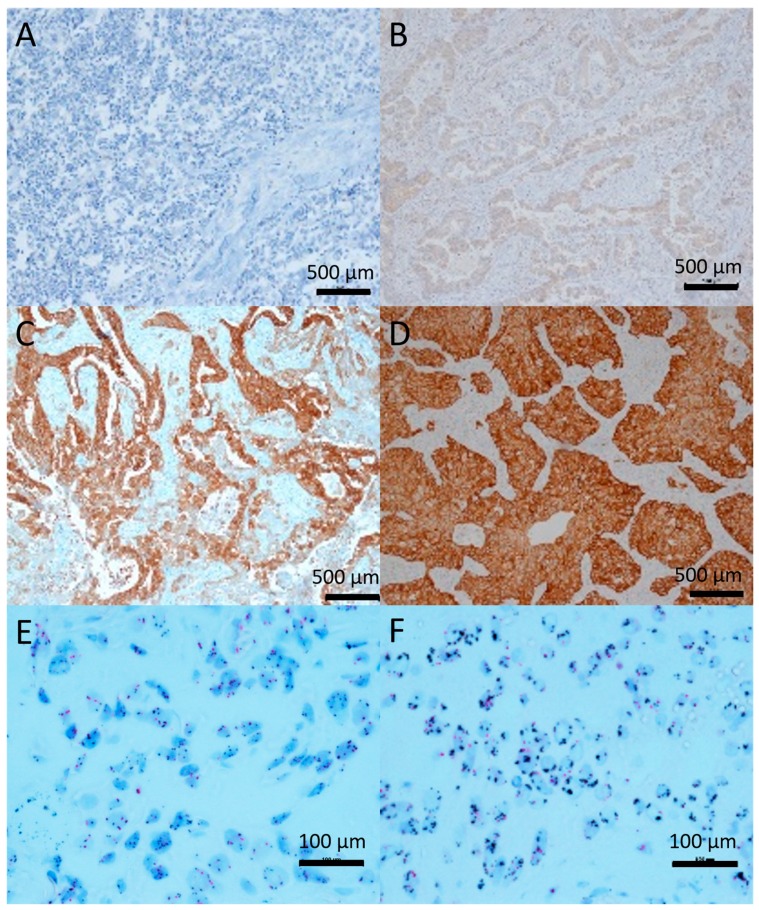
Representative images of c-MET-IHC (SP44) and c-MET CISH (MET DNP/CEN7 DIG). Images A–D are at 400× magnification. Images E-F are on 600× magnification. (**A**) tumor with no c-MET expression (0). (**B**) tumor with low expression (1+). (**C**) tumor with moderate (2+) expression, showing a very heterogeneous staining. (**D**) tumor with high (3+) expression. (**E**) tumor showing low c-MET amplification, ratio c-MET/centromere 7 is 2. (**F**) tumor showing high c-MET amplification with c-MET gene foci present, ratio: 4.54. Scale bar = 1000 µm.

The 3 µm slides were stained with the ready-to-use anti-total c-Met (SP44) rabbit monoclonal antibody (9.75 µg/mL antibody) (Ventana, Basel, Switzerland) or the ready-to-use anti-total EGFR (3C6) mouse monoclonal antibody (3 mg/mL antibody, Ventana, Basel, Switzerland), as exemplified by the representative images in Figure 3. Chromogenic in situ hybridization (CISH) was performed with the MET DNP (40 µg/mL) and CEN7 DIG (8 µg/mL) probes (Ventana, Basel, Switzerland) on 5 µm slides (Figure 3). All stainings were performed according to the manufacturer’s instructions on a Ventana Benchmark Ultra. For c-Met-IHC, internal controls were used (endothelium = 1+; bronchial epithelium = 2+). Both the intensity and number of positive cells were determined [50]: <50% of cells, low intensity = 0; >50% of cells, low intensity or <50% median intensity = 1+; >50% median intensity or <50% high intensity = 2+; >50% high intensity = 3+. For the in situ hybridization, 10 cells each in 5 fields per sample were counted [15]. The ratio between c-Met copies and the centromere of chromosome 7 was calculated. The cut-off for amplification was a ratio of 2. The scoring of the EGFR-IHC was performed as the c-Met-IHC scoring. External controls were included on every slide: normal lung, prostate, placenta and thyroid.

### 4.3. EGFR-Mutation Analysis

The DNA was isolated with a Reliaprep FFPE gDNA tissue kit (Promega, Leiden, Netherlands). High-resolution melting (HRM) was performed [58]. 2 µL of PCR product was purified with Exosap-it (Affymetrix, High Wycombe, United Kingdom) and sequenced with the BigDye Terminator v1.1 V Cycle Sequencing Kit (LifeTechnologies, Merelbeke, Belgium) on the ABI Prism 3130 Genetic Analyzer (LifeTechnologies, Merelbeke, Belgium).

### 4.4. Next Generation Sequencing

A library containing all exons of c-Met and TP53, including known mutation hot-spots, was prepared with the custom designed TPME Multiplex amplification of Specific Targets for Resequencing (MASTR^TM^) assay (Multiplicom, Niel, Belgium) with MID for Illumina Miseq (Illumina, Eindhoven, The Netherlands).with a MiSeq Reagent Kit v2 (500 cycles).

### 4.5. Data Analysis

An in-house annotation and filtering tool VariantDB [59] (available online: http://www.biomina.be/app/variantdb/) was used to identify Single Nucleotide Variants (SNV). The NM_000245.2 sequence was used for c-Met, while NM_000546.5 was used for TP53 sequences. Mapping on crystal structures was performed with Yasara view (yasara.org). Statistical analysis was performed with SPSS (version 23, IBM, NY, USA) using the Chi^2^-test, Kaplan-Meier test, log-rank Mantel-Cox and Cohen’s kappa test.

## 5. Conclusions

In conclusion, this study revealed a strong correlation between EGFR expression and EGFR mutations, as well as TP53 mutations and c-Met expression, in therapy-naïve primary resection samples. Secondly, we found a moderate agreement in the c-Met status between the primary resection tissues and the paired metastases. Finally, we identified a new mutation in the ligand-binding domain of c-Met, I333T, which might influence the binding of antibodies targeting the HGF-binding domain. This evidence supports the notion that c-Met represents a primary potential target in NSCLC, also demonstrating a high clinical relevance in the treatment with EGFR-TKIs, since previous research showed that mutations in the c-Met receptor might cause innate resistance to antibodies [54].

## Figures and Tables

**Table 1:** List of c-Met mutations in the NSCLC specimens. Abbreviations, NA: Not available; SNP: single nucleotide polymorphism; MAF: minor allele frequency; IHC: immunohistochemistry.

Mutation	RefSNP	Allelic Balance (%)	MAF	Reads on Position	c-Met-IHC	Histology
N375S	rs33917957	61	2.3%	10230	1+	Adeno
E168D	rs55985569	50	0.5%	4659	3+	Adeno
S203T	rs200861145	12	0.1%	2093	2+	Squamous
S203T	rs200861145	11	0.1%	2709	2+	Adeno
S203T	rs200861145	15	0.1%	5209	1+	Squamous
S203T	rs200861145	14	0.1%	4590	2+	Squamous
S203T	rs200861145	20	0.1%	1816	2+	Adeno
E168D	rs55985569	43	0.5%	9341	3+	Adeno
I333T	NA	8	NA	7765	3+	Squamous
G783E	NA	11	NA	1061	3+	Squamous
S203T	rs200861145	45	0.1%	6009	2+	Adeno
C3082+1G>T	rs869320707	12	0%	1353	2+	Adeno
C2942−1G>A	NA	39	NA	1180	3+	Adeno

**Table 2:** List of TP53 mutations, and the associated c-Met amplifications and EGFR mutations in the NSCLC specimens. Abbreviations, NA: Not available; WT: wild type.

Sample ID	WT Codon	Mutant Codon	p.Mutant	c.Mutant	Functionality	c-Met	EGFR
8	TGC	TTC	p.C242F	c.725G>T	Partial function Non-functional	3+	WT
9	GGG	TGG	p.G334W	c.1000G>T	Partial function	1+	WT
10	CGC	CTC	p.R337L	c.1010G>T	Non-functional	2+	WT
17	AGG	ATG	p.R249M	c.746G>T	Non-functional	3+	WT
18	CCT	TCT	p.P278S	c.832C>T	Non-functional	2+	L858R
19	GTC	GAC	p.V157D	c.470T>A	Non-functional	2+	WT
58	GAA	CAA	p.E258Q	c.772G>C	Partial function	1+	NA
59	AGG	ACG	p.R249T	c.746G>C	Non-functional	2+	NA
72	CGC	CTC	p.R175L	c.524G>T	Partial function	0	NA
74	CGA	TGA	p.R342X	c.1024C>T	NA	2+	NA
75	AGA	GGA	p.R280G	c.838A>G	Partial function Non-functional	1+	NA
79	GAC	CAC	p.D281H	c.841G>C	Non-functional	0	WT
80	GAG	AAG	p.E285K	c.853G>A	Non-functional	2+	NA
81	GCC	CCC	p.A276P	c.826G>C	Non-functional	2+	NA
85	GGG	GTG	p.G334V	c.1001G>T	Partial function	3+	NA
92	CGC	CAC	p.R158H	c.473G>A	Non-functional	3+	NA
94	CGT	CTT	p.R273L	c.818G>T	Non-functional	1+	NA
97	AGA	GGA	p.R280G	c.838A>G	Non-functional	2+	L858R
97	GGA	GTA	p.G266V	c.797G>T	Non-functional	2+	L858R
97	GGC	TGC	p.G245C	c.733G>T	Non-functional	2+	L858R
101	CGG	CAG	p.R267Q	c.800G>A	Partial function	0	NA
101	CCT	TCT	p.P190S	c.568C>T	Partial function	0	NA
103	GGT	GTT	p.G262V	c.785G>T	Non-functional	1+	NA
104	CGT	CCT	p.R273P	c.818G>C	Non-functional	3+	L858R
105	AAG	AGG	p.K132R	c.395A>G	Partial function Non-functional	1+	NA
117	GGA	GTA	p.G266V	c.797G>T	Non-functional	1+	NA
118	GAG	TAG	p.E294X	c.880G>T	NA	0	NA
121	CCT	ACT	p.P278T	c.832C>A	Non-functional	1+	NA
128	GTG	GGG	p.V216G	c.647T>G	Non-functional	2+	NA
131	CGA	TGA	p.R196X	c.586C>T	NA	0	NA
133	CCC	TCC	p.P151S	c.451C>T	Non-functional	0	NA
136	GAG	TAG	p.E298X	c.892G>T	NA	2+	NA
137	CAT	CGT	p.H179R	c.536A>G	Partial function Non-functional	2+	NA
141	CAT	CGT	p.H214R	c.641A>G	Non-functional	2+	WT
149	CGT	CTT	p.R273L	c.818G>T	Non-functional	3+	WT
153	CAG	TAG	p.Q192X	c.574C>T	NA	2+	L858R
160	AGA	GGA	p.R280G	c.838A>G	Partial function Non-functional	2+	WT

**Table 3:** Patient data.

Age	Range: 36–78 Years (Mean: 62)	N
**Histology**	Adenocarcinoma	104
	Squamous carcinoma	38
	Large cell (or not otherwise specified, NOS)	11
**Differentiation**	Well	32
	Moderate	63
	Poor	26
**Invasiveness**	Non-invasive	49
	Invasive	102
**Gender**	Male	109
	Female	44
**Smoking**	Non-smoker	61
	Smoker	92

## Supplementary Materials

The following are available online at https://www.mdpi.com/1420-3049/24/24/4443/s1, Figure S1: Kaplan-Meier survival curves of NSCLC patients grouped according to c-Met expression, as assessed by IHC, Figure S2: Overlay of the three-dimensional structure of the wild-type (WT) Sema domain of c-Met (chain B of PDB ID 4K3J, in cyan-green) with the predicted structure of mutant I333T of the Sema domain of c-Met (magenta), as displayed with pymol version 2.2.0 (The PyMOL Molecular Graphics System, Version 2.0 Schrödinger, LLC).
