# Supplementary material for: The Role of c-Met as a Biomarker and Player in Innate and Acquired Resistance in Non-Small-Cell Lung Cancer: Two New Mutations Warrant Further Studies
Source: Molecules. 2019 Dec 4;24(24):4443. doi: 10.3390/molecules24244443 (PMC6943481; doi:10.3390/molecules24244443)
Supplement: Supplementary file 1 [file molecules-24-04443-s001.pdf]

Supplementary Material

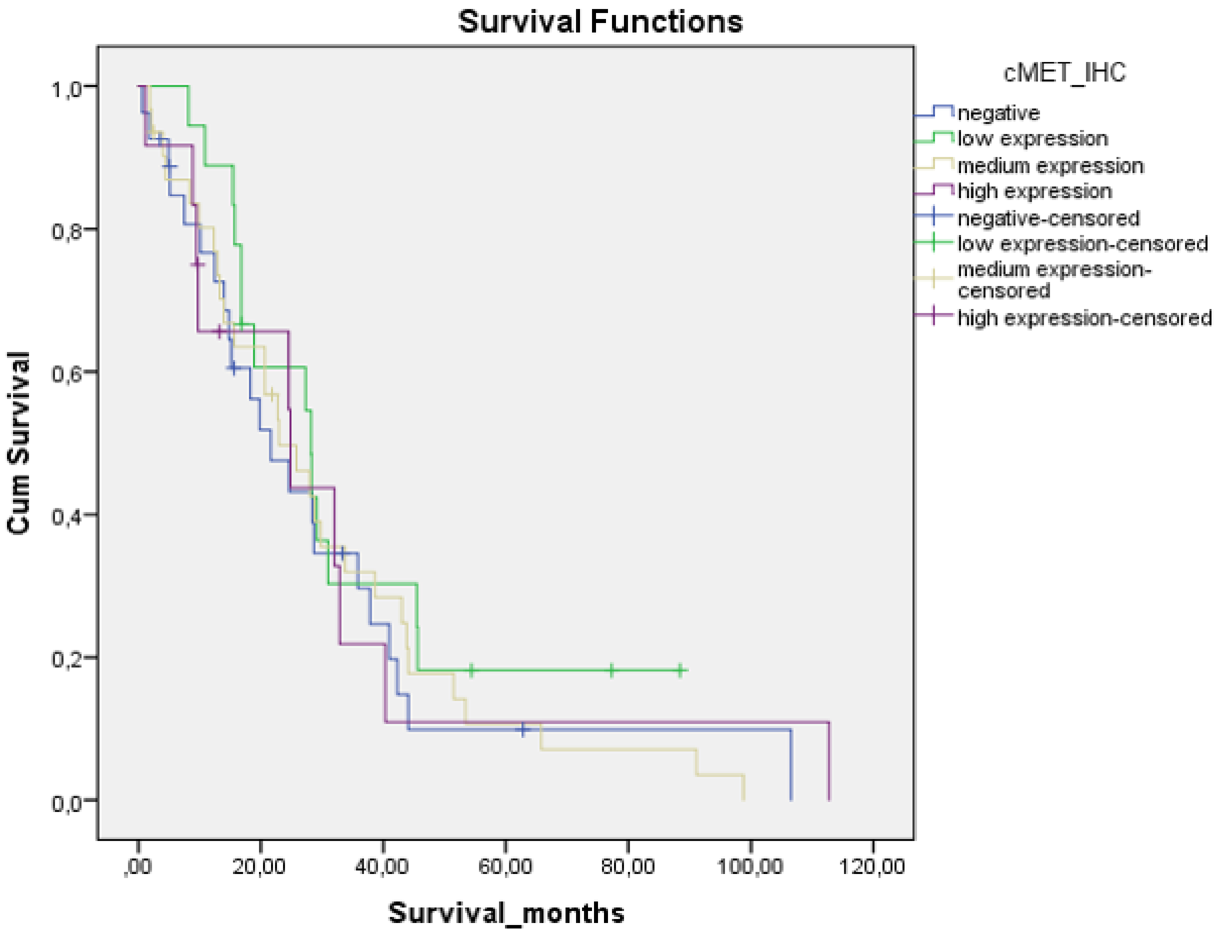

Supplementary Figure S1. Kaplan-Meier survival curves of NSCLC patients grouped according to c-Met expression, as assessed by IHC.

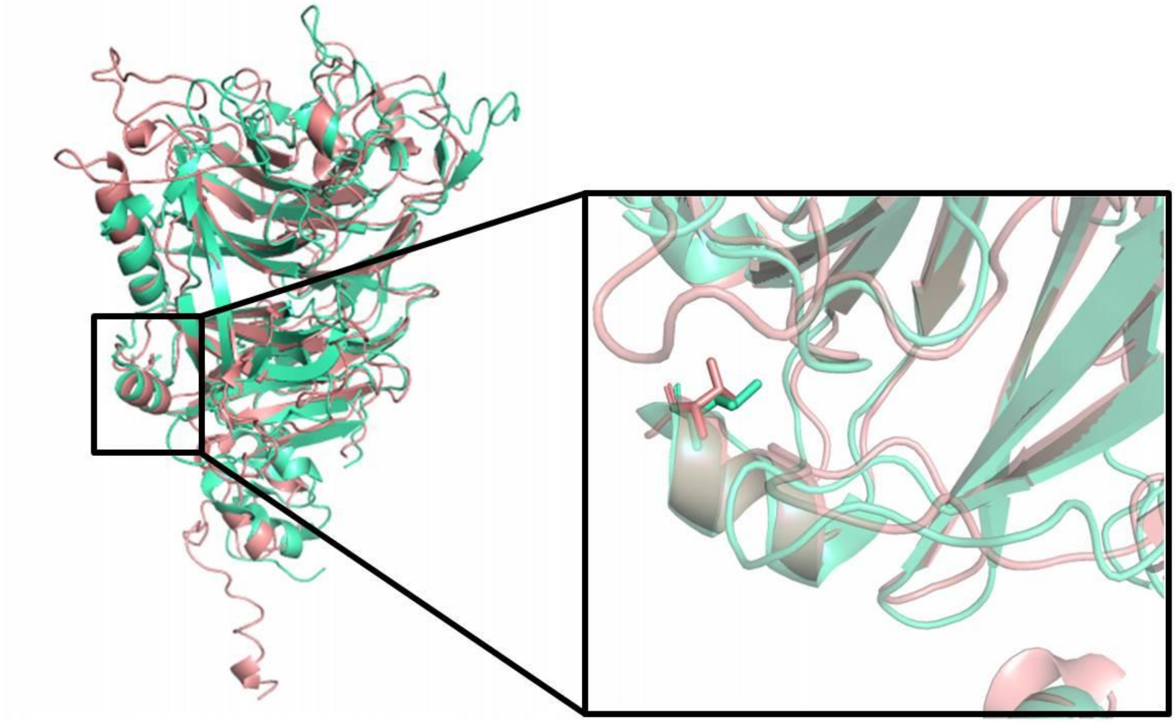

Supplementary Figure S2. Overlay of the three-dimensional structure of the wild-type (WT) Sema domain of c-Met (chain B of PDB ID 4K3J, in cyan-green) with the predicted structure of mutant I333T of the Sema domain of c-Met (magenta). This overlay and the zoom-in into the region of residue 333 (with residue 333 shown in stick representation) shows that except for the C-terminal flexible loop of c-Met's Sema domain, the three-dimensional structure of the interface region with onartuzumab in the 4K3J crystal structure (cf. panel C of Figure 2; in which the Sema domain of c-Met is similarly oriented) is not substantially affected. The structure of mutant I333T was predicted using the I-TASSER webserver, as described earlier (supplemental references 1-3). (https://zhanglab.ccmb.med.umich.edu/I-TASSER, accessed on 18 November 2019).

Supplemental references:

1. A Roy, A Kucukural, Y Zhang. I-TASSER: a unified platform for automated protein structure and function prediction. Nature Protocols, 5: 725-738 (2010).
2. J Yang, R Yan, A Roy, D Xu, J Poisson, Y Zhang. The I-TASSER Suite: Protein structure and function prediction. Nature Methods, 12: 7-8 (2015).
3. J Yang, Y Zhang. I-TASSER server: new development for protein structure and function predictions. Nucleic Acids Research, 43: W174-W181 (2015).
